# Urine Leak From the Necrotic Lower Pole of a Transplanted Kidney: A Rare Complication in a Pediatric Deceased Donor Kidney Transplant Recipient

**DOI:** 10.7759/cureus.13613

**Published:** 2021-02-28

**Authors:** Ronit Patnaik, Muhammad U Rabbani, Elizabeth Thomas, Gregory A Abrahamian

**Affiliations:** 1 General Surgery, University of Texas Health Science Center at San Antonio, San Antonio, USA; 2 Transplant Surgery, University of Alabama at Birmingham, Birmingham, USA; 3 Transplant Surgery, University of Texas Health Science Center at San Antonio, San Antonio, USA

**Keywords:** urine leak, deceased donor kidney transplant, renal artery thrombosis, necrotic kidney

## Abstract

Kidney transplant patients are prone to a variety of complications, even for the most experienced surgical teams. Our busy transplant center recently performed its 5,000th solid organ transplant. We present the case of an 18-year-old male with end-stage renal disease who underwent a deceased donor kidney transplant. He developed a urine leak from the necrotic lower pole of his graft kidney and subsequently developed urosepsis and was admitted. Clinicians must have a high suspicion for complications in the immediate post-operative period in kidney transplant patients. In this report, we will highlight our diagnostic and treatment steps to preserve the patient’s graft while addressing his rare complications.

## Introduction

Patients are prone to a variety of complications after a kidney transplant. Infarction of the lower pole of a transplanted kidney, however, is extremely rare. In the case series by Nehoda et al. involving 1,600 renal transplants, only one case of renal pole infarction was noted [1]. Historically, this complication was treated by transplant nephrectomy [2]. Furthermore, urine leak rates occur in 1-3.5% of kidney transplant recipients [3]. Here, we report the case of urine leak from the necrotic lower pole of a transplanted kidney and our subsequent management.

## Case presentation

We present the case of an 18-year-old male with end-stage renal disease secondary to focal segmental glomerulosclerosis who presented to the transplant service; the patient had been on peritoneal dialysis for six months. The donor was a male in his mid-30s with a kidney donor profile index of 13% and positive for Epstein-Barr virus and cytomegalovirus. The patient underwent a deceased donor kidney transplant with induction by Thymoglobulin and Solumedrol. His graft functioned appropriately and his post-operative day 1 renal ultrasound, as per protocol, was normal. He was discharged after an uneventful six-day hospital stay.

One week later, the patient presented to the clinic with abdominal pain, fluid leakage from his incision, and increased urinary frequency. He was admitted and diagnosed with a urine leak and urinoma via a workup including a computed tomography of the abdomen/pelvis and renal scintigraphy as seen in Video 1 and Video 2, respectively.

**Video 1:** CT from patient's second admission when he presented with a urine leak. CT, computed tomography

**Video 2:** Nuclear medicine imaging of the kidney (renal scintigraphy) from his second admission: brisk urinary leak from the renal pelvis into the necrotic lower pole of the renal transplant.

**Figure 1:**
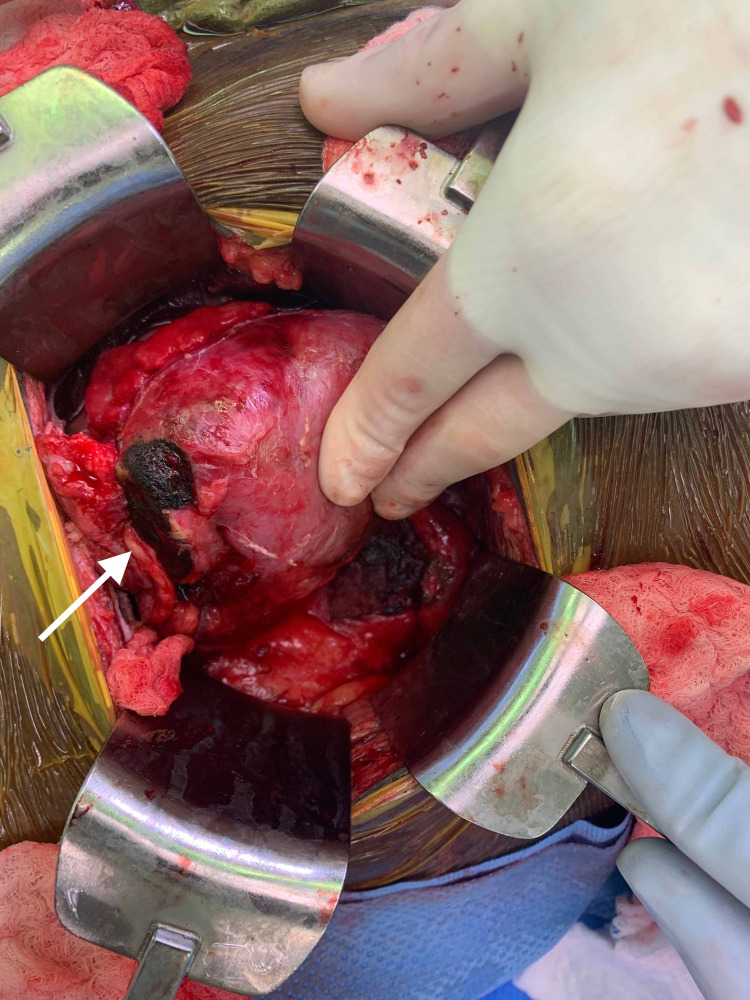
Intra-operative view: necrotic inferior pole (white arrow).

**Figure 2:**
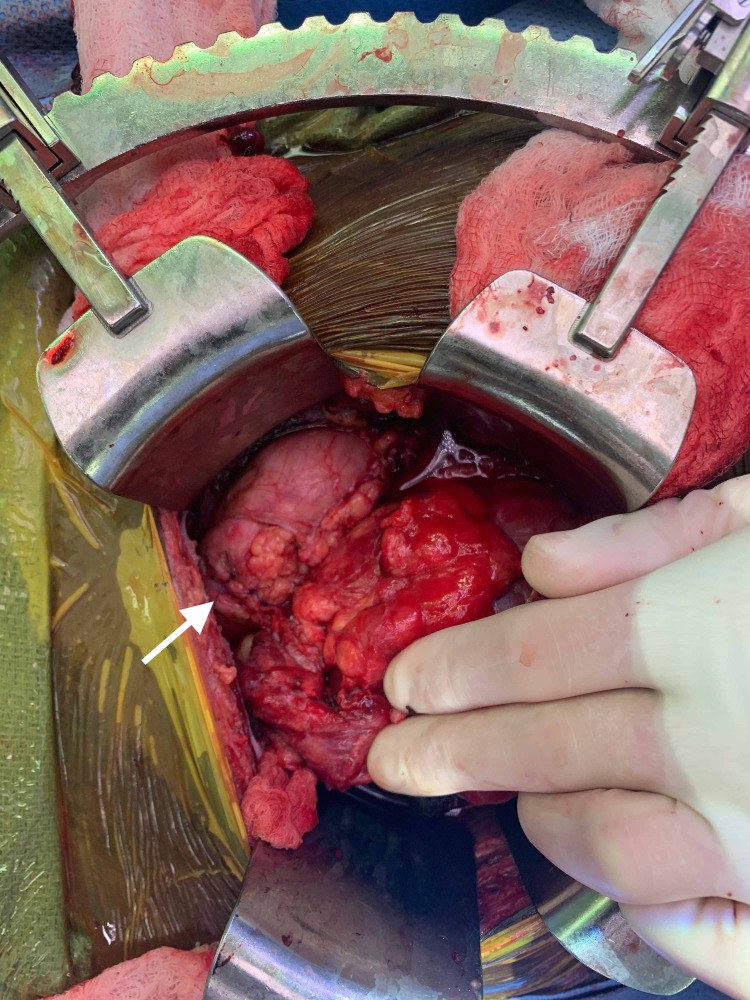
Intra-operative view: after partial nephrectomy of the ischemic inferior pole of the transplanted kidney (white arrow).

The patient subsequently underwent operative exploration for the urinary leak. During the operation, he underwent a partial nephrectomy of the ischemic inferior pole along with ureteral reconstruction via a ureteroneocystostomy with a perinephric Jackson-Pratt (JP) drain left in place (Figures 1, 2).

During his hospital stay, the patient continued to have a high urine output (>2 L) from his perinephric JP drain, suggesting a persistent urine leak. A few days later, the patient underwent cystoscopy with cystogram with pediatric urology. He was, yet again, found to have a urinary leak from the remnant lower pole of the transplanted kidney. At this point, the plan to address the urine leak was drainage via the Foley, double J ureteral stent, and JP drain; they were to be left in place for two months of decompression. The patient was then discharged home. His JP output gradually decreased during this time, suggesting an improvement in the urine leak.

**Figure 3:**
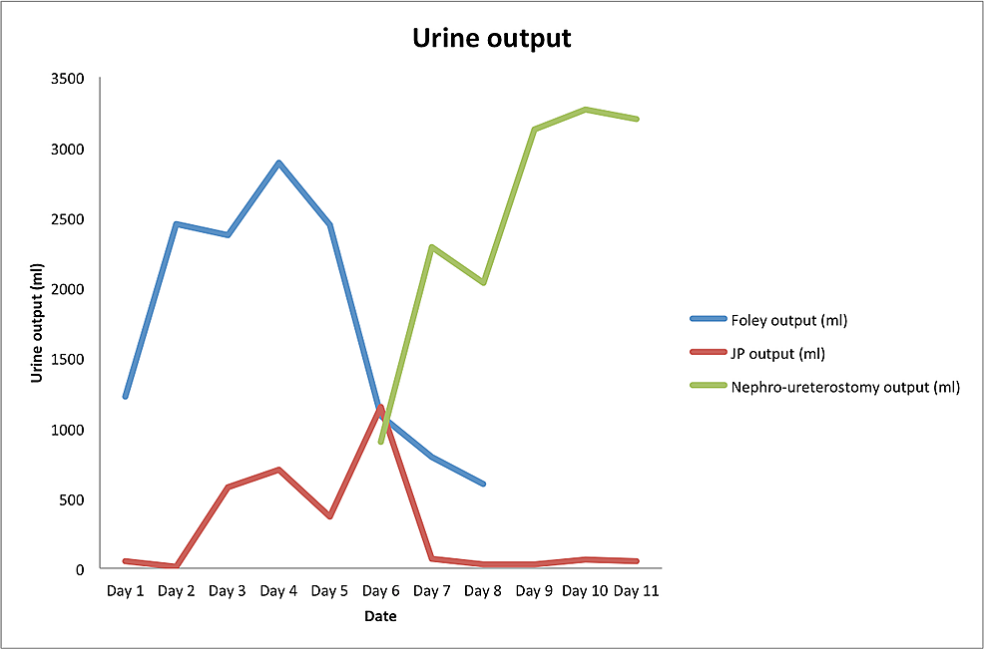
Urine output through various drainage sites (blue: Foley; red: JP drain; green: nephro-ureterostomy stent/catheter). JP, Jackson Pratt Nephro-ureterostomy stent/catheter placed on day six. Foley removed on day 8 The days are defined as days of admission on the patient's third hospitalization.

Unfortunately, two weeks later, the patient returned to the hospital with *Enterococcus faecalis* urosepsis. He was started on vancomycin and cefepime and transitioned to a two-week course of ampicillin once susceptibilities developed; ampicillin was converted to oral amoxicillin prior to discharge. During his stay, he continued to have increased JP drain output and an elevated creatinine in the JP output, which was concerning for a worsening urinary leak. He was placed on broad-spectrum antibiotics and the source of his infection was deemed to be an infected urine leak. The patient then had a percutaneous nephrostomy placed with a nephro-ureteral stent, and during this procedure, a urine leak was noted from the remnant lower pole of the transplanted kidney. His drain outputs during this third hospitalization are showcased in Table 1 and Figure 3.

**Table 1:** Urine output from various drainage sources, including Foley, JP drain, and nephro-ureterostomy catheter/stent. JP, Jackson Pratt The days are defined as days of admission on the patient's third hospitalization

Day	Foley output (mL)	JP output (mL)	Nephro-ureterostomy output (mL)
Day 1	1,220	50	
Day 2	2,450	10	
Day 3	2,375	575	
Day 4	2,890	700	
Day 5	2,445	370	
Day 6	1,085	1,150	900
Day 7	790	65	2,285
Day 8	600	25	2,035
Day 9		25	3,125
Day 10		60	3,265
Day 11		50	3,200

**Figure 4:**
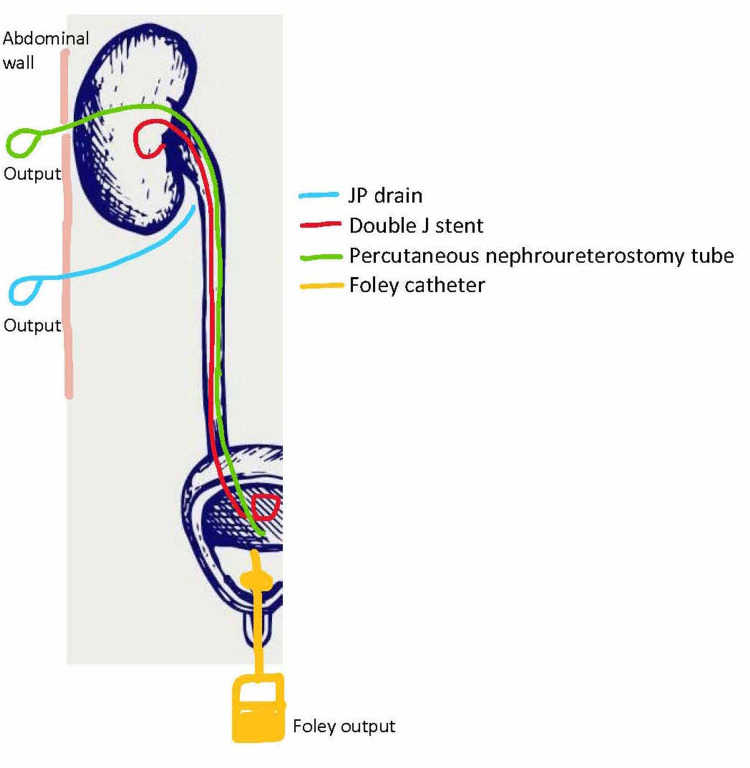
Urinary tract decompression. Green: Nephro-ureterostomy tube; blue: JP drain; red: double J stent; yellow Foley catheter. JP, Jackson Pratt

The nephro-ureteral stent allowed another path for urinary decompression and led to a decrease in his JP drain output (urine leak) (Figures 3, 4). His Foley and double J stent were subsequently removed on day 8 and day 10, respectively; hence, at this point, decompression was primarily performed by the nephro-ureteral stent. After his urine leak resolved, the nephro-ureteral stent was removed, approximately five months after the original complication.

## Discussion

Our patient’s kidney transplant was performed in the busiest transplant center in South Texas. The university transplant center has performed over 5,000 solid organ transplants since 1970. The most common complications after a kidney transplant are hemorrhage, vascular thrombosis, urologic complications, lymphoceles, and infections [3,4]. An inferior pole infarction in a transplanted kidney is a rare complication. In fact, in the case series by Nehoda et al. involving 1,600 renal transplants, only one case of renal pole infarction was noted [1]. This complication likely occurs secondary to a post-operative thrombotic event in an inferior sub-segmental renal artery [5].

Our patient had just one renal artery and an additional inferior pole sub-segmental renal artery was likely not appreciated. It is well documented in the surgical literature that renal grafts with multiple renal arteries are associated with a higher risk of complications and delayed graft function, but they have comparable long-term outcomes for the graft and patient survival [5]. The major urologic complications after kidney transplants include leak, obstruction, and stenosis. Urine leaks occur in 1-3.5% of kidney transplant recipients and the most common location is the ureterovesical anastomosis [3]. This can be avoided by preserving the distal peri-ureteral fatty tissue during preparation of the kidney for transplant. Routine use of double J stents at the time of ureterovesical anastomosis appears to decrease the rate of major urologic complications [6].

The St. Barnabas review of urologic complications in renal transplant patients showed a urine leak rate of 0.61% [7]. In their practice, they treated small urine leaks with a double J stent and Foley drainage; for larger leaks, they recommended immediate ureteral reconstruction with consideration for nephrostomy drainage, as needed. We used these principles, along with a nephro-ureteral stent, to decompress the urinary system and to allow the site of the urine leak to heal.

In a case report by Nehoda et al. (1998), an infarcted lower pole in a transplanted kidney occurred secondary to intra-renal sub-segmental artery occlusion [1,8]. Their treatment was partial nephrectomy along with primary closure of the collecting system. Historically, the treatment of an infarcted segment of a transplanted kidney was graft nephrectomy [1,2,8]. Nephron-sparing surgery is a well-established treatment for a variety of benign and malignant disease. Similar to Nehoda et al., our goal was to save the patient’s graft while avoiding the morbidity of a graft nephrectomy.

## Conclusions

Historically, the treatment for an infarcted segment of the transplanted kidney was a transplant nephrectomy. With our experience, we recommend that at least one attempt should be made at excision of the necrotic portion of the graft (nephron-sparing nephrectomy) and salvaging an otherwise functioning graft. The treatment for urine leak continues to be adequate drainage and decompression.
